# Effect of social media-based education on self-care status, health literacy, and glycated hemoglobin in patients with type 2 diabetes

**DOI:** 10.3389/fpubh.2025.1507726

**Published:** 2025-01-20

**Authors:** Ali Safdari, Nazi Nejat, Abdollah Abolfathi, Fatemeh Mehrabi, Fatemeh Rafiei

**Affiliations:** ^1^Department of Nursing, Malayer School of Medical Sciences, Chronic Diseases (Home Care) Research Center, Hamadan University of Medical Sciences, Hamadan, Iran; ^2^Department of Nursing, Arak University of Medical Sciences, Arak, Iran; ^3^Iranian Social Security Organization, Arak, Iran; ^4^Department of Biostatistics, Faculty of Medical Sciences, Arak University of Medical Sciences, Arak, Iran

**Keywords:** HbA1c, self-care, health literacy, type 2 diabetes, social networks, Iran

## Abstract

**Objectives:**

This study examines the impact of social media-based education on health literacy status, self-care, and glycosylated hemoglobin (HbA1c) in individuals with type 2 diabetes (T2D).

**Method:**

This educational intervention study was carried out from March 2022 to June 2022 on diabetic patients visiting the diabetes clinic in Arak, Iran. The patients split into two groups: the virtual education group (*n* = 38) using the Telegram messaging platform and the control group (*n* = 38). Patients in the virtual education group received multimedia messages about T2D daily for 4 weeks. Data analysis utilized SPSS version 23 and statistical tests.

**Results:**

The results of this study showed that the overall score of health literacy and the dimensions of reading, understanding, and evaluation were significantly higher in the intervention group than in the control group (*p* < 0.05). However, the score for the access dimension did not show a significant difference between the two groups (*p* > 0.05). The Wilcoxon test results indicated that the average HbA1c score significantly decreased in the intervention group before and after the intervention. However, these changes were not significant in the control group. Nevertheless, the Mann–Whitney test did not indicate a significant statistical difference between the groups regarding the average HbA1c score before and after the intervention (*p* > 0.05).

**Conclusion:**

The findings of this study suggest that social networks provide a suitable platform for delivering self-care education to individuals with T2D. Furthermore, in the long term, it might positively impact the patients’ HbA1c levels. Future studies with larger sample sizes can be beneficial in this area.

## Introduction

Diabetes is recognized as one of the most common chronic diseases worldwide (1). The World Health Organization (WHO) even refers to diabetes as a silent epidemic (2). It is predicted that the number of individuals affected by this disease will exceed 592 million by 2035 (3). The prevalence of diabetes is steadily increasing in various countries worldwide (4). Statistics in Iran also show that diabetes affects 7% of the country’s population, and with the current trend, experts estimate that this number will reach 6 million by 2030 (5). Type 2 diabetes (T2D) is the most prevalent form of diabetes globally, accounting for approximately 90% of diabetes cases (6).

Uncontrolled diabetes is associated with the onset of disabilities, cardiovascular diseases, nephropathy, neuropathy, retinopathy, and patient mortality (7). Nevertheless, diligent monitoring and control of the disease can significantly delay or even prevent the onset of diabetes-related complications (6). Any care activity requires patient involvement, and self-care is considered a key component of diabetes management (8). The collection of behaviors adopted by individuals with diabetes or at risk of its complications, enabling them to manage the disease independently, is referred to as self-care activities (7). Proper adherence to self-care activities leads to benefits such as adequate blood sugar control, reduced complications, decreased hospitalizations, and improved quality of life (9). Previous studies have shown that most individuals with diabetes have limited self-care capabilities (10, 11).

In recent years, patients have become significantly more interested in taking responsibility for their disease management, but they need education (11). Therefore, education plays a crucial role in enhancing the self-care abilities of individuals with diabetes (5). Given the chronic and lifelong nature of the disease, self-care for these patients is a lengthy, complex, and challenging process (12). It requires significant lifestyle changes (13). This underscores the growing importance of self-care education (14). Despite their inherent potential, in-person educational sessions have limitations, including impractical transportation for low-income groups, time and location constraints, and a lack of human and financial resources (15).

Recent advancements in information and communication technologies have created opportunities to facilitate learning through multimedia virtual education (16). Social media platforms, in particular, play a significant role in the prevention, care, and management of chronic diseases (8). Their effectiveness in managing various chronic diseases has been well documented (17).

Despite the numerous benefits offered by various social messaging platforms, limited studies have explored their impact on managing type 2 diabetes in developing countries, revealing a gap between the use of these platforms and the understanding of the disease’s management (17). More than three-quarters of individuals with diabetes in low- to middle-income countries face significant challenges in accessing adequate healthcare and treatment (18). According to the World Health Organization, fewer than 50% of patients in these countries benefit from self-care activities (19).

A systematic review and meta-analysis conducted in 2021 also indicated that the self-care score of Iranian diabetic patients is approximately 48.86%, which falls short of the desired level. Based on this finding, the researchers strongly recommend developing interventions aimed at improving self-care among individuals with diabetes (11). The findings of another study in Pakistan demonstrate gaps in individuals with diabetes knowledge and highlight the importance of self-care education. The study also identifies obstacles such as financial constraints and excessive work commitments that hinder patients’ ability to engage in self-care (6).

Obstacles such as high costs, insufficient access to medication, and unequal distribution of healthcare services across different regions are among the challenges faced by developing countries (7, 17). Among the factors affecting the prevention and control of diabetes is having sufficient awareness of the disease, the factors affecting its occurrence and how to prevent this disease. One of the factors that greatly affects the level of awareness and, as a result, more effective control and prevention of the disease, diabetes is health literacy, which is the degree to which individuals have the capacity and ability to acquire, process and understand information related to health and the services they need to make appropriate decisions about their health (9).

Given the direct correlation between health literacy levels and self-care activities (5), we have undertaken the design and implementation of a study on self-care education in virtual spaces to investigate its effects on self-care status, health literacy, and HbA1c levels among individuals with T2D in Iran.

## Method

### Participants

This educational intervention study was conducted on patients attending the diabetes clinic in Arak, Iran, from March 2022 to June 2022. The sampling method used in this study random sampling’. After acquiring the necessary permissions, eligible individuals were selected from among patients attending the clinic. They were introduced to the research objectives, and upon obtaining their informed consent, they were enrolled in the study.

The inclusion criteria for this study were as follows: a definitive diagnosis of T2D by a specialist endocrinologist, an age range of 30–60 years, a minimum of 6 months of diabetes history, ownership of a smartphone, the ability to use social messaging apps such as Telegram, the absence of psychological disorders, literacy in reading and writing, and Iranian nationality. The exclusion criteria included unwillingness to continue participation, pregnancy, and hospitalization during the study.

The required sample size was calculated based on a similar study, with an effect size of 0.7, *α* = 0.05, and *β* = 0.1, resulting in a total sample size of 34 participants per group (20). Considering the attrition rate (10%), 38 individuals were enrolled in each group. The power of the study was calculated to be 80%, ensuring an adequate sample size to detect meaningful differences.

### Intervention

In this study, patients were randomly allocated to two groups: the intervention group (*n* = 38) and the control group (*n* = 38). After explaining the study and creating a channel on the Telegram messaging platform, the lead author invited all participants to join by sending them an invitation link. Access to this messaging platform was ensured for all participants.

Necessary self-care instructions from credible and up-to-date sources were shared daily through the channel. The content, provided in Persian, included topics related to self-care, such as physical activity, blood glucose monitoring, types of diabetes, dietary guidelines, diabetes complications, and foot care. To ensure participant engagement with the study material, the researcher monitored daily attendance in the channel, tracked the number of views for each message, and contacted any participant who had not accessed the channel for more than 48 h to inquire about the reasons. If a participant failed to engage, they were excluded from the study. The instructional program lasted for 4 weeks. This step was done by the researcher.

Patients in the control group did not receive any educational materials during the study. To adhere to ethical principles, an educational package was provided to these patients after the completion of the study.

### Instruments

The researchers developed the self-care questionnaire used in this study by reviewing relevant literature and studies on diabetes. The items in the self-care section of the questionnaire allowed participants to report their self-care activities and the factors affecting their self-care behaviors over the past 7 days. The questionnaire consisted of two sections. The first section included demographic information (age, gender, marital status, education level, occupation, and social media usage) and clinical conditions (duration of diabetes, type of treatment received, and family history of diabetes). The second section contained 16 questions related to diabetes self-care behaviors, compliance diet, exercise, monitoring, treatment, and prevention of complications.

The face validity of the questionnaire was established by presenting it to 10 faculty members with expertise in questionnaire design at Arak University of Medical Sciences. The Cronbach’s alpha coefficient for this questionnaire was 0.91, indicating good internal consistency.

Health literacy was assessed using the Health Literacy for Iranian Adults (HELIA) questionnaire, designed by Montazeri et al. (21), which has demonstrated desirable validity and reliability. The Cronbach’s alpha values for the dimensions of the HELIA questionnaire ranged from 0.72 to 0.89, confirming the reliability of the various dimensions of the instrument (21).

The questionnaire included 33 items assessing patients’ health literacy across five dimensions: access (6 questions), reading (4 questions), understanding (7 questions), evaluation (4 questions), and decision-making and application of health information (12 questions). All items, except those in the reading dimension, were designed on a 5-point Likert scale: (Always: 5, Most of the time: 4, Sometimes: 3, Rarely: 2, Not at all: 1). The items in the “reading skills” dimension used a modified Likert scale: (Very Easy: 5, Easy: 4, Neither easy nor difficult: 3, Difficult: 2, Very Difficult: 1).

The raw score for each participant in each dimension was calculated by summing their responses on each item. To determine the total score, the sum of all item scores was divided by the number of dimensions (5). Scores ranging from 0 to 50 were considered inadequate, scores from 50.1 to 66 were categorized as semi-sufficient, scores from 66.1 to 84 were considered sufficient, and scores from 84.1 to 100 were classified as excellent (22). Participants completed the instrument for evaluation through self-reporting at both the beginning and end of the intervention.

### Glycosylated hemoglobin (HbA1c)

In this study, HbA1c was assessed through laboratory testing at the beginning and end of the intervention. All tests were conducted in a centralized laboratory for all patients.

### Data analysis

Data analysis was performed using descriptive statistics, Wilcoxon test, Fisher’s exact test, analysis of covariance (ANCOVA), and the Mann–Whitney *U* test to compare differences between groups. Data analysis was conducted using SPSS version 23, with a significance level set at 0.05.

### Ethical considerations

The design and execution of this research adhered to the Helsinki Declaration. Before beginning the study, all participants were informed about the study’s objectives and were informed that they could withdraw from the study at any time without affecting their relationship with medical professionals or caregivers. The present study has been registered with the Ethics Committee in Research at Arak University of Medical Sciences under the reference number IR.ARAKMU.REC.1398.042.

## Results

Two patients withdrew from the study due to a lack of interest, and one patient did not complete the questionnaire. Additionally, three participants were excluded due to inadequate responses on the Telegram messaging platform. Consequently, data analysis was performed with 70 participants. Of these participants, 38.6% were male, and 15.7% resided in rural areas. The demographic characteristics of the study participants are presented in Table 1.

**Table 1:** Distribution of individual characteristics of the patients in intervention and control groups.

Variable	Intervention group (*n* = 35)	Control group (*n* = 35)	*p*-value
Gender (*n*, %)			0.806^a^
Male	13 (37.14)	14 (40)	
Female	22 (62.86)	21 (60)	
Education (*n*, %)			0.496^a^
Elementary school	12 (34.29)	10 (28.57)	
Middle school	7 (20)	7 (20)	
High school	7 (20)	13 (37.14)	
University	9 (25.71)	5 (14.29)	
Marital status (*n*, %)			0.356^a^
Single	8 (22.86)	5 (14.29)	
Married	27 (77.14)	30 (85.71)	
Age (*n*, %)			0.907^a^
30–40	8 (22.86)	9 (25.71)	
41–50	14 (40)	12 (34.29)	
51–60	13 (37.14)	14 (40)	
Location (*n*, %)			0.513^b^
City	31 (88.57)	28 (80)	
Village	4 (11.43)	7 (20)	
BMI (*n*, %)			0.330^b^
Less than 18.5	1 (2.86)	3 (8.57)	
18.5–24.9	18 (51.43)	13 (37.14)	
25–29.9	15 (42.86)	19 (54.29)	
30–34.9	1 (2.86)	0 (0.0)	
Income (*n*, %)			0.508^a^
High-income	7 (20)	9 (25.71)	
Middle-income	18 (51.43)	20 (57.14)	
Low-Income	10 (28.57)	6 (17.14)	

Notes: Abbreviations: no, number; SD, standard deviation; BMI, body mass index.

*p < 0.05 was considered statistically significant.

^aChi-square tests.^

^bFisher’s exact test.^

The results indicated that there was no statistically significant difference between the mean scores of reading, access, understanding, evaluation, decision-making, and overall health literacy scores in the two groups before the intervention (*p* > 0.05). However, after the intervention, the mean scores for reading, understanding, evaluation, decision-making, and total health literacy in the intervention group were significantly higher than in the control group (*p* < 0.05). The post-intervention access score did not show a statistically significant difference between the two groups (*p* > 0.05) (Table 2).

**Table 2:** Comparison of the mean and standard deviation of health literacy in two groups before and after the intervention.

		Before	After	Difference in score before and after	Test	
		Mean ± SD	Mean ± SD	Mean ± SD	Statistics	*p*-value
Reading	Intervention	53.21 ± 34.17	68.92 ± 28.17	−15.71 ± 30.48	−2.719	0.007
	Control	51.78 ± 25.3	54.64 ± 28.73	−2.85 ± 30.81	−0.432	0.666
Statistics		599	434			
*P*-value		0.872	0.031			
Access	Intervention	54.76 ± 31.95	67.85 ± 29.07	−13.09 ± 27.71	−2.515	0.012
	Control	51.78 ± 24.55	54.64 ± 28.23	−2.85 ± 30.71	−0.599	0.549
Statistics		599	595.5			
*P*-value		0.872	0.067			
Understanding	Intervention	56.63 ± 31.46	69.79 ± 27.73	−13.16 ± 27.51	−2.521	0.012
	Control	51.02 ± 27.05	56.93 ± 28.71	−5.91 ± 34.07	−1.017	0.309
Statistics		555.5	423			
*P*-value		0.499	0.024			
Evaluation	Intervention	56.07 ± 29.56	70.71 ± 28.63	−14.64 ± 29.15	−2.733	0.006
	Control	53.03 ± 25.33	54.82 ± 29.08	−1.78 ± 38.54	−0.115	0.909
Statistics		572	409			
*P*-value		0.624	0.015			
Decision-making	Intervention	46.36 ± 30.13	65.17 ± 28.03	−18.80 ± 23.94	4.648	<0.001
	Control	50.35 ± 30.92	48.09 ± 29.46	2.26 ± 44.22	−0.303	0.764
Statistics		−0.546	2.485			
*P*-value		0.587	0.015			
Total (health literacy)	Intervention	52.07 ± 28.82	67.77 ± 26.81	−15.69 ± 22.78	−3.655	<0.001
	Control	51.25 ± 21.27	52.77 ± 26.25	−1.51 ± 31.76	−0.328	0.743
Statistics		606	387			
*P*-value		0.939	0.008			

The results revealed that the changes in the mean scores for reading, access, understanding, evaluation, decision-making, and total health literacy in the intervention group were statistically significant and showed an increase (*p* < 0.05) (Table 2).

According to the results, there was no statistically significant difference in the mean scores of the self-care questionnaire (follow the diet, physical activity, treatment, control, and prevention of complications) between the control and intervention groups before the intervention (*p* > 0.05). However, after the intervention, the scores for control and prevention of complications differed significantly between the groups, with the intervention group showing higher scores than the control group (*p* < 0.05). The average scores for the other dimensions did not show statistically significant differences after the intervention (*p* > 0.05) (Table 3).

**Table 3:** Comparison of the mean and standard deviation of self-care in two groups before and after the intervention.

		Before	After	Difference in score before and after	Test	
		Mean ± SD	Mean ± SD	Mean ± SD	Statistics	*P*-value
Follow the Diet	Intervention	61.79 ± 16.97	57.46 ± 8.52	4.32 ± 16.06	−1.59	0.12
	Control	61.67 ± 16.39	60.16 ± 12.11	1.51 ± 18.92	−0.472	0.640
Statistics		0.031	−1.07			
*P*-value		0.976	0.286			
Physical activity	Intervention	49.38 ± 25.09	56.32 ± 24.37	−6.93 ± 39.35	−1.05	0.290
	Control	57.0 ± 25.07	66.93 ± 22.27	−9.93 ± 23.56	−2.4	0.016
Statistics		−1.271	543			
*P*-value		0.208	0.06			
Treatment	Intervention	35.11 ± 16.57	36.68 ± 19.32	−1.56 ± 7.55	−1.33	0.183
	Control	38.06 ± 18.21	37.41 ± 18.03	0.64 ± 10.91	−0.654	0.513
Statistics		546	589			
*P*-value		0.356	0.732			
Control	Intervention	43.71 ± 14.56	86.57 ± 12.7	−42.85 ± 17.33	−5.092	<0.001
	Control	48.57 ± 11.28	61.42 ± 26.13	−12.85 ± 28.31	−2.397	0.017
Statistics		486	226			
*P*-value		0.134	< 0.001			
Prevention of complications	Intervention	60.05 ± 19.52	77.25 ± 15.65	−17.20 ± 22.98	−3.654	<0.001
	Control	70.43 ± 17.36	64.13 ± 17.19	6.29 ± 28.26	−1.347	0.178
Statistics		422	337			
*P*-value		0.025	0.001			
Total (self-care)	Intervention	52.39 ± 10.6	60.51 ± 6.8	−8.11 ± 11.85	−3.359	0.001
	Control	56.72 ± 8.26	56.88 ± 5.56	−0.16 ± 10.62	−0.017	0.986
Statistics		435.5	339.5			
*P*-value		0.038	0.019			

The results indicated that the changes in the average scores for control and prevention of complications in the intervention group were statistically significant and increased (*p* < 0.05). However, in the control group, the changes in the overall self-care score dimensions were not significant (*p* > 0.05) (Table 3).

The results showed a significant difference in the mean total score of the self-care questionnaire between the two groups (*p* < 0.05). To control for confounding variables, analysis of covariance (ANCOVA) was conducted on the baseline self-care total score, revealing a significant difference in the mean self-care total score after the intervention between the two groups, with higher scores observed in the intervention group (*p* < 0.05). Additionally, the changes in the total self-care score in the intervention group were both statistically significant and increasing (*p* < 0.05) (Table 3).

The indicated no statistically significant difference in the mean HbA1c levels before and after the intervention between the groups (*p* > 0.05). However, the Wilcoxon test results showed that the mean HbA1c levels in the intervention group statistically significantly (*p* < 0.05). This difference was not statistically significant in the control group (Table 4).

**Table 4:** Comparison of the mean and standard deviation of HbA1c in two groups before and after intervention.

		Before	After	Difference in score before and after	Test	
		Mean ± SD	Mean ± SD	Mean ± SD	Statistics	*P*-value
HbA1c	Intervention	7.65 ± 1.42	7.45 ± 1.34	0.2 ± 0.52	−2.36	0.018
	Control	7.93 ± 1.46	7.93 ± 1.33	0.005 ± 0.42	−0.533	0.594
*P*-value		0.553	0.145			

The results of the correlation analysis indicated that changes in the scores of self-care dimensions did not have a statistically significant relationship with changes in the scores of health literacy dimensions in either group (*p* > 0.05) (Table 5).

**Table 5:** Relationship between changes in health literacy level scores and changes in self-care scores in the intervention group.

	Prevention of complications		Control		Treatment		Physical activity		Diet		Total self-care score	
	Correlation	*P*-value	Correlation	*P*-value	Correlation	*P*-value	Correlation	*P*-value	Correlation	*P*-value	Correlation	*P*-value
Reading	0.004	0.980	0.205	0.238	−0.079	0.651	0.212	0.221	0.186	0.284	0.190	0.273
Access	−0.008	0.966	0.180	0.301	−0.150	0.390	0.089	0.610	0.226	0.192	0.149	0.392
Understanding	0.074	0.671	0.192	0.269	−0.105	0.547	0.159	0.360	0.159	0.360	0.188	0.279
Evaluation	0.133	0.446	0.144	0.409	−0.102	0.561	0.136	0.435	0.108	0.538	0.176	0.311
Decision-making	0.008	0.965	0.231	0.183	0.013	0.943	−0.017	0.923	−0.175	0.315	−0.051	0.770
Total health literacy	0.042	0.812	0.233	0.179	−0.084	0.632	0.110	0.531	0.071	0.686	0.120	0.493

## Discussion

This study aimed to investigate the impact of social media-based education on self-care, health literacy, and glycated hemoglobin levels in individuals with type 2 diabetes. The findings of this study demonstrated that education through social networks effectively enhances health literacy among diabetic patients.

Initially, no statistically significant difference was found in health literacy scores between the intervention and control groups. However, after receiving the education, the intervention group showed significant improvements in reading, understanding, evaluation, decision-making, behavior, and overall health literacy scores. In contrast, the control group did not exhibit such improvements. These findings are consistent with previous studies highlighting the potential of online interventions in enhancing health literacy among individuals with chronic diseases (23, 24).

Health literacy refers to the knowledge and skills necessary to make informed health decisions. It plays a crucial role in managing chronic diseases such as diabetes. Low health literacy is associated with poorer adherence to treatment, worsening medical conditions, increased hospitalization, and higher healthcare costs (25). A meta-analysis has shown that health literacy has a small but significant effect on glycemic management in patients (26). It is important to note that effective self-care in patients is closely linked to adequate health literacy (25).

Our study findings emphasize the effectiveness of virtual education through the Telegram messaging app in improving patients’ health literacy. A cross-sectional study by Moulaei et al. (13) demonstrated that Iranian users obtain more information about their health conditions through social media platforms such as WhatsApp, Telegram, and Instagram. Based on these findings, it is recommended to incorporate social media-based education into routine care programs for individuals with T2D.

Our study findings demonstrated that the mean total score of the self-care questionnaire in the intervention group was significantly higher than that in the control group. These findings are supported by previous studies. Biglar Chopoghlo et al. (17) also showed that self-care education on social media platforms is associated with increased self-efficacy scores in Iranian adolescent girls with type 1 diabetes. Similarly, Alanzi et al. (20) found that using the WhatsApp social network led to a significant increase in knowledge and self-efficacy among T2D patients in the intervention group compared to the control group. Tang et al. (23) reported in a cross-sectional study that the use of innovative technologies, such as mobile phones and multimedia tools, is effective in improving self-care activities in individuals with T2D. These results indicate that social networks should be considered a valuable tool for education due to their benefits, such as accessibility, ease of use, access to up-to-date health information, cost reduction, and the possibility of online interactions in educational programs (13).

In our study, although the overall score of the self-care questionnaire after the intervention was significantly higher in the social media education group compared to the control group, the scores of some self-care dimensions, including physical activity and treatment, in the control group also showed significant improvement after the study, compared to the beginning of the study.

This finding may be attributed to the self-care methods or education received through physicians and other communication channels in the control group. Additionally, this result highlights the complexity of the relationship between health literacy and self-care in diabetic patients. Further research is needed to clarify the influential factors and potential confounding variables in self-care among T2D patients. Existing evidence also supports this finding. For example, Hosseinzadeh et al. (16) demonstrated in their study that both virtual and face-to-face education led to increased self-care in pregnant women with gestational diabetes, compared to the control group. However, no significant difference was found between the two intervention groups.

Aligholipour et al. (19) in a similar study conducted in Iran, demonstrated that although both social network-based and face-to-face education led to increased scores in patients’ self-care activities, no significant difference in outcomes was found between the two intervention groups. Despite the potential effectiveness of education through modern technologies, such as mobile phones, being comparable to face-to-face education (16), researchers emphasize that the interpersonal relationship between the nurse and the patient is a fundamental principle of nursing care. Therefore, virtual education should not entirely replace face-to-face education (19). On the other hand, using virtual networks presents risks, such as exposure to incorrect information, misinterpretation of medical results, and distractions caused by advertisements on these platforms (13).

The findings of the present study indicate that changes in self-care dimension scores do not have a statistically significant relationship with changes in health literacy scores, which contradicts the findings of İlhan et al. (25). Maleki Chollou et al. (27) also demonstrated in their study that there is a positive and almost direct relationship between health literacy dimensions and self-care behaviors in T2D patients across all dimensions. In contrast with our findings, Eyüboğlu and Schulz (28) found no association between health literacy and self-care behaviors in diabetes patients. This discrepancy may be attributed to differences in the implementation methods of educational interventions, measurement tools, and target groups. Some existing evidence also links patients’ demographic characteristics to their self-care activities (23). Education remains an integral part of the care of patients with T2D (29).

İlhan et al. (25) demonstrated in their study that individuals with low health literacy have difficulties in self-care, understanding health information, and adhering to treatment. Therefore, early identification of knowledge deficiencies regarding self-care can lead to better treatment adherence and delay in the onset of complications (29). Social networks allow individuals to utilize educational resources with lower cost and without time and location constraints, while expanding their social interactions (17). RobatSarpooshi et al. (5) also reported an average level of self-care scores in Iranian patients in a study aimed at investigating the relationship between health literacy and self-care behaviors. Furthermore, the results of this study showed that increasing levels of education and awareness among patients were associated with increased adherence to self-care behaviors. The findings of these studies indicate that developing an educational program, especially a self-care program, can be effective in enhancing patient self-efficacy.

To our surprise, we did not find any statistically significant relationship between changes in self-care behaviors and changes in HbA1C levels in the intervention group. This finding contradicts some previous studies that reported a positive correlation between improved self-care behaviors and HbA1C levels (25). Nevertheless, the results of some studies support our findings. Lee et al. demonstrated in a study that assessed the effectiveness of self-care education using a mobile phone program in T2D patients that changes in HbA1c levels between the control and intervention groups at week 26 were not significant (30). Contradictory results regarding the impact of self-care behaviors observed in different countries may be influenced by socio-economic and cultural differences among these countries and the variable levels of self-care status in individuals with diabetes. Additionally, differences in follow-up periods and duration of interventions are other potential reasons for this discrepancy. In our study, although a significant difference in the mean HbA1c levels between the intervention and control groups was not observed, HbA1c levels decreased after the intervention. Some studies have reported significant improvements in blood glucose control in diabetic patients with online interventions, while others have reported limited or no effect (19). Possible reasons for this difference in reported results may include the variability in follow-up durations and different laboratory methods for measuring patients’ blood glucose levels.

One of the strengths of this study is its examination of the impact of social media-based education on health literacy and self-care in individuals with T2D. However, there are several limitations to this study. First, self-care is a complex construct that may be influenced by various confounding factors. For instance, diabetic patients have access to multiple sources of disease-related education, which could potentially affect the study outcomes. Additionally, the relatively short duration of the follow-up and the intervention represents another limitation. Therefore, it is recommended that future research investigate the long-term effects of virtual education on glycemic control and the prevention of diabetes-related complications, with extended follow-up periods. Also, some variables such as personal motivation, family support have an effect on patient self-care that were not measured in this study. Therefore, it is recommended that studies be conducted with the exclusion of these confounding factors.

## Conclusion

The results of this study have shown that social networks, such as Telegram, can effectively provide self-care education to a large number of participants, offering broader and more accessible education compared to individual, in-person methods. Given the limited number of healthcare providers in developing countries, this approach is cost-effective, as it does not impose a significant financial burden when compared to other traditional teaching methods. Additionally, considering the varied work schedules and personal commitments of participants, this platform eliminates the time constraints typically associated with in-person education.

## Data Availability

The raw data supporting the conclusions of this article will be made available by the authors, without undue reservation.
